# The epidemiology of delayed HIV diagnosis in Ibadan, Nigeria

**DOI:** 10.1177/09564624221106523

**Published:** 2022-06-30

**Authors:** Michael O Oluwalana, Olutosin A Awolude, Zhiwei Gao, Peter K Daley

**Affiliations:** 1 Clinical Epidemiology Unit, Faculty of Medicine, Division of Community Health and Humanities Faculty of Medicine, 7512 Memorial University, St John’s, Canada; 2 University College Hospital, Ibadan, Nigeria; 3 APIN Public Health Initiative, Nigeria

**Keywords:** Nigeria, human immunodeficiency virus, delayed diagnosis, prevention of transmission, risk factors, trends in annual cases

## Abstract

**Background:**

Human immunodeficiency virus infection (HIV) is one of the major health burdens in Nigeria. Delayed HIV diagnosis remains a significant driver of HIV transmission. The risk factors of delayed HIV diagnosis have not been widely studied in Nigeria. This observational study examined demographic risk factors for delayed HIV diagnosis and the trends in the annual total cases of delayed HIV diagnosis in Ibadan, Nigeria.

**Methods:**

We examined the data on HIV patients enrolled in care at the University College Hospital’s Antiretroviral Therapy (ART) clinic in Ibadan, Nigeria. Delayed HIV diagnosis was defined as a Cluster of Differentiation 4 (CD4) count of less than 350 cells/mm³ at the time of diagnosis. The association between delayed HIV diagnosis and risk factors was analyzed using logistic regression. The trends in the annual total cases of delayed HIV diagnosis over time were examined.

**Results:**

This study included 3458 HIV patients. There were 1993/3458 prevalent cases of delayed HIV diagnosis (57.6%). The risk factors for delayed HIV diagnosis were older age, retirement, marriage separation, never married, and widowed female. The factors that were significantly associated with a low risk of delayed HIV diagnosis were student and tertiary education. There was a progressive decline in the annual cases of delayed HIV diagnosis.

**Conclusions:**

Although the cases of delayed HIV diagnosis are still high, they are declining. Human immunodeficiency virus testing should be targeted at populations at risk of delayed diagnosis. Considerable public awareness and education programs about HIV testing may significantly reduce delayed HIV diagnosis in Nigeria.

## Introduction

Since the pandemic began, an estimated 76.1 million people have been infected with Human Immunodeficiency Virus (HIV), with 35 million deaths from AIDS-related diseases.^1^ Globally, 36.7 million people were living with HIV at the end of 2018, and it is estimated that Sub-Saharan Africa accounted for 70% of this burden.^2,3^ Nigeria has the second-largest HIV epidemic globally.^4^ In 2018, there were 130,000 new cases of HIV and 53,000 HIV-related deaths in Nigeria.^5^ Heterosexual transmission accounts for over 90% of HIV transmissions in Nigeria.^2^ The 2019 prevalence of HIV in Nigeria among adults aged 15–49 years is 1.4% compared to 2.8% in 2017, and the estimated number of people living with HIV (PLHIV) has decreased from 3.1 million in 2017 to 1.9 million in 2019.^6,7^ It is estimated that 47% of PLHIV in Nigeria are diagnosed, 96% are on antiretroviral therapy (ART), and 81% have achieved viral suppression on ART.^8^ As a result, Nigeria has not achieved the United Nations Program on HIV/AIDS (UNAIDS) 90-90-90 treatment target that was proposed in 2014.^9^

Delay in HIV diagnosis represents a missed opportunity to prevent transmission through viral suppression.^10^ The European consensus definition of delayed diagnosis is a CD4 count of less than 350 cells/mm³ or clinical AIDS or the presence of opportunistic infection at the time of diagnosis.^11^ Several developed countries have reported a prevalence of delayed HIV diagnosis ranging between 14.9% and 55.9%.^12^ Although the prevalence of delayed HIV diagnosis is not reported nationally in Nigeria, some observational studies exist. Agaba et al. (2014) observed an 85.6% prevalence of delayed diagnosis among 14,000 PLHIV in Jos, Nigeria.^12^ A cross-sectional study in Nigeria observed a mean time between HIV infection and diagnosis was 6.7 years and 8.1 years for men and women, respectively.^13^ In Nigeria and the United Kingdom, delayed HIV diagnosis is associated with increased mortality and decreased survival time.^14,15^

Demographic factors have been described as predictors of delayed HIV diagnosis.^16^ In most countries, older age, heterosexuality, men who have sex with men (MSM), and injecting drug users (IVDU) were all associated with a delayed HIV diagnosis.^17^ Male gender, older age, being a civil servant, widowed or divorced, unemployment, poverty, and fear of discrimination have been previously described as risk factors associated with delayed HIV diagnosis in Nigeria.^12^ Identifying these risk factors is a critical first step in developing strategies toward targeting HIV testing. The objectives of this study were to describe risk factors associated with delayed HIV diagnosis in Nigeria and to examine the trends in the annual total cases of delayed HIV diagnoses over time.

## Methods

### Study Design, Setting and Population, Data Source

Antiretroviral therapy (ART)-naïve HIV patients with documented CD4 count at diagnosis who attended the ART clinic at the University College Hospital, Ibadan, Nigeria for the first time and enrolled in care between October 2013 and December 2018 were included. Patients' information was collected at the first clinic visit using a standardized preassessment form and entered into the APIN Public Health Initiatives database. While the data included ART-naïve HIV patients with their first known CD4 count, the HIV tests or CD4 tests may not be the first tests for some patients as some may had previous tests during their routine doctors’ visits without linkage to care.

We made secondary use of data made available by the APIN Public Health Initiatives database. APIN Public Health Initiative is a non-governmental organization (NGO) in Nigeria and a direct implementing partner of the United States Centers for Disease Control and Prevention (CDC) with a focus on HIV control and prevention and other current and emerging public health issues.^18^ APIN Public Health Initiative collaborates closely with the federal government of Nigeria, major STI clinics in several states of Nigeria, and relevant stakeholders to strengthen policy and service delivery systems for HIV treatment and prevention.^18^

Inclusion criteria were ART-naïve HIV patients with documented CD4 count at diagnosis and aged 15 years or older. Of the 3559 patients available, 101 (2.8%) were excluded (58 no CD4 count, 30 missing data, 13 age less than 15). Ethics approval was given by the APIN Institutional Review Board (IRB) in Nigeria and the Health Research Ethics Board (HREB) of Newfoundland and Labrador, Canada. Both ethics review boards determined that the de-identified secondary data we used in this study did not require consent.

### Analysis

We defined delayed HIV diagnosis in accordance with European consensus as a CD4 count of less than 350 cells/mm³ at the time of diagnosis.^11^ All variables were categorical, with the exception of the CD4 count at diagnosis, which was a continuous variable. Differences in percentage for categorical variables and means for continuous variable were examined by t-test and Chi-squares tests. Logistic regression analysis was performed to identify risk factors associated with delayed HIV diagnosis in Nigeria. The predictor (independent) variables, which were socio-demographic factors of the patients, were gender, age at diagnosis, employment status, marital status, educational status, occupation, leader in a religious organization, and reference categories were selected. The outcome variable is delayed HIV diagnosis. All analyses were conducted using the SAS System for Windows (copyright 2019 SAS Institute Inc).

### Logistic regression

Independent variables that were significant at *p* ≤ .20 level in the univariate analysis were included in the multivariate model. Effect modification was also included. The strength of association between the risk factors and outcome was reported as odds ratios, 95% confidence interval and *p*-value. In addition to the logistic regression analysis, the Cochran-Armitage linear trend test was used to determine the relationship between the outcome and the ordinal independent variables (age group and level of education).

### Annual total cases of delayed HIV diagnosis

Annual observations of the total delayed HIV diagnosis were compared annually between 2014 to 2018.

## Results

### Descriptive statistics

Table 1 describes the included PLHIV. The percentage of delayed HIV diagnosis was 1993/3458 (57.6%).

**Table 1.** Descriptive analysis.

Variables	Labels	Total (%) (*N* = 3458)
Delayed HIV	Yes	57.6
	No	42.4
Age	15–39 years	55.7
	≥40 years	44.3
Gender	Male	34.0
	Female	66.0
Marital status	Single	25.0
	Married	52.6
	Separated	8.7
	Divorced	1.9
	Widowed	11.8
Educational level	None	16.4
	Primary	19.8
	Secondary	36.5
	Tertiary	27.3
Occupation	Trader	37.4
	Student	7.2
	Commercial driver	5.3
	Civil servant	3.0
	Retiree	1.7
	Other	45.4
Employment status	Employed	97.9
	Unemployed	2.1
Leader in a religious organization	Yes	1.1
	No	98.9
Enrollment year	2013	6.3
	2014	25.9
	2015	19.1
	2016	19.3
	2017	15.7
	2018	13.8

### Univariate analysis

The univariate analysis is summarised in Table 2. The odds of delayed HIV diagnosis in PLHIV aged 40 years or more was higher than those aged 15–39 years (OR 1.73; 95% CI 1.51–1.99), higher in males than females (OR 1.50; 95% CI 1.30–1.73), higher among separated PLHIV (OR 2.29; 95% CI 1.74–3.02), divorced PLHIV (OR 1.85; 95% CI 1.09–3.15), and widowed PLHIV (OR 1.36; 95% CI 1.09–1.70) compared to married PLHIV, higher among patients who were leaders in a religious organization (OR 2.47; 95% CI 1.17–5.22) than those who were not, higher among retirees (OR 4.20; 95% CI 2.50–8.61), and higher among commercial drivers (OR 2.28; 95% CI 1.61–3.23), compared to other occupations. The odds of delayed HIV diagnosis were lower among single PLHIV compared to married PLHIV (OR 0.84; 95% CI 0.72–0.99), lower among PLHIV with a tertiary education compared to no formal education (OR 0.64; 95% CI 0.52–0.79) and lower among students (OR 0.50; 95% CI 0.38–0.66) compared to other occupations.

**Table 2.** Univariate analysis of delayed HIV diagnosis.

Variable	% Of delayed HIV diagnosis	Odds ratio	95% Cl		*p*-value	Type 3 *p*-value
			Lower	Upper		
Age						
≥40 years	65.01	1.73	1.51	1.99	<.0001	
15–39 years	51.77	1				
Gender						
Male	64.06	1.50	1.30	1.73	<.0001	
Female	54.32	1				
Marital status						
Single	51.68	0.84	0.72	0.99	.0385	<0.0001
Separated	74.42	2.29	1.74	3.02	<.0001	
Divorced	70.15	1.85	1.09	3.15	.0065	
Widowed	63.33	1.36	1.09	1.70	.0065	
Married	55.94	1				
Educational level						
Primary	65.06	1.24	0.99	1.56	.0673	<0.0001
Secondary	59.03	0.96	0.78	1.17	.6864	
Tertiary	48.94	0.64	0.52	0.79	<.0001	
None	60.04	1				
Occupation						
Trader	59.00	1.11	0.96	1.29	.1717	<0.0001
Student	39.52	0.50	0.38	0.66	<.0001	
Commercial driver	74.73	2.28	1.61	3.23	<.0001	
Civil servant	56.73	1.01	0.68	1.51	.9571	
Retiree	84.48	4.20	2.05	0.96	<.0001	
Other	56.46	1				
Employment status						
Unemployed	58.33	1.03	0.64	1.65	.9037	
Employed	57.62	1				
Leader in a religious organization						
Yes	76.92	2.47	1.17	5.22	.0177	
No	57.41	1				

### Multivariate analysis

The multivariate analysis is summarised in Table 3. The odds of a delayed HIV diagnosis were significantly higher in PLHIV aged 40 years or older than in those aged 15–39 years (OR 1.29; 95% CI 1.10–1.51). The linear trend test revealed a linear relationship between delayed HIV diagnosis and age categories (15–40, 21–40, 41–60, and 61 years or more) (trend test: *p* < .0001), indicating that the probability of delayed HIV diagnosis increases with increasing age. The odds of a delayed HIV diagnosis were significantly lower among PLHIV with tertiary education compared to those with no formal education (OR 0.71; 95% CI 0.56–0.88). There was a linear relationship between delayed HIV diagnosis and educational level (trend test: *p* < .0001), indicating that the probability of delayed HIV diagnosis decreases as educational level increases from none to tertiary education. In comparison with other occupations, the odds of delayed HIV diagnosis were higher among retirees (OR 3.24; 95% CI 1.56–6.74) and lower among students (OR 0.61; 95% CI 0.45–0.82). We observed a statistically significant interaction between gender and married status (*p* = .0249). The odds of delayed HIV diagnosis were higher among male PLHIV who were separated compared to married male PLHIV (OR 2.16; 95% CI 1.21–3.88). The odds of delayed HIV diagnosis were higher among single female PLHIV (OR 1.36; 95% CI 1.09–1.71), separated female patients (OR 2.17; 95% CI 1.57–2.99), and widowed female PLHIV (OR 1.37; 95% CI 1.06–1.77) compared to married female patients.

**Table 3.** Multivariate analysis of delayed HIV diagnosis.

Variables	Odds ratio	95% Cl		*p*-value	Type 3 *p*-value
		Lower	Upper		
Age					
≥40 years	1.29	1.10	1.51	.0016	
15–39 years	1				
Educational status					
Primary	1.18	0.93	1.50	.1706	<0.0001
Secondary	1.01	0.82	1.24	.9614	
Tertiary	0.71	0.56	0.88	.0021	
None	1				
Occupation					
Trader	1.09	0.92	1.29	.3018	<0.0002
Student	0.61	0.45	0.82	.0012	
Commercial driver	1.35	0.93	1.97	.1151	
Civil servant	1.10	0.73	1.67	.6466	
Retiree	3.4	1.56	6.74	.0017	
Other	1				
Interaction between gender and marital status					
Male					
Single	0.77	0.57	1.04	.0857	0.0249
Separated	2.16	1.21	3.88	.0096	
Divorced	2.02	0.67	6.11	.2122	
Widowed	0.93	0.54	1.61	.7952	
Married	1				
Female					
Single	1.36	1.09	1.71	.0073	
Separated	2.17	1.57	2.99	<.0001	
Divorced	1.73	0.93	3.24	.0856	
Widowed	1.37	1.06	1.77	.0179	
Married	1				

### Trends in the annual total cases of delayed HIV diagnosis

Table 4 summarised the trends in the annual total cases of delayed HIV diagnosis. Between 2014 and 2018, the number of cases of delayed HIV diagnosis steadily decreased. Over a 4-years period, delayed HIV diagnosis cases decreased by 5%.

**Table 4.** Trends in the annual total cases of delayed HIV diagnosis.

Year of diagnosis	Cases of HIV		Delayed HIV diagnosis	
	*n* (3247)	% (100)	*n* (886)	% (58.1)
2014	903	27.8	495	15.2
2015	660	20.3	383	11.8
2016	667	20.5	397	12.2
2017	542	16.7	313	9.6
2018	476	14.7	297	9.1

## Discussion

Early diagnosis of HIV with immediate initiation of ART and retention in care will not only result in viral suppression and reduced mortality but also reduce the risk of HIV transmission.^19,20^ Describing factors associated with delayed HIV diagnosis may inform targeted HIV screening. Older PLHIV were more likely to have delayed HIV diagnosis compared to younger PLHIV, and this is consistent with previous studies in Nigeria and other parts of the world^12,17,21^ Older PLHIV may be perceived as a low risk group.^22^ Association of delayed HIV diagnosis and male gender, and delayed HIV diagnosis and marital status have been well documented in many studies.^12,21,23,24,25^ Separated male and female PLHIV were found to be associated with delayed HIV diagnosis, with more delayed diagnosis among females. Married women are less likely to have a delayed HIV diagnosis, which may be linked to routine mandatory screening for HIV offered to pregnant women in Nigeria during their first antenatal visit.^12,26^ The differential effect of marital status on delayed HIV diagnosis based on gender may be explained by unmarried women’s lower health care utilization compared to married women.^26^

People living with HIV who had a tertiary education are less likely to have a delayed HIV diagnosis compared to PLHIV with no formal education; this finding is consistent with a previous study.^27^ People with higher education are often financially independent and are empowered to make well-informed decisions related to their health and wellbeing.^28,29^ We found a high risk of delayed HIV diagnosis among retirees, which has not been previously reported, and a low risk among students. Retirees and older people are among the most vulnerable populations in Nigeria due to the lack of a national plan for social welfare for senior citizens resulting in a low standard of living and diminished health status.^30,31^ Poor pension programs and insufficient health insurance coverage with resultant difficulty to access health care services in Nigeria^30^ may explain the high risk of delayed HIV diagnosis observed among retiree and older age group. Several targeted screening programs have been conducted among students in various Nigerian higher educational institutions.^32,33^ This countrywide awareness among students in Nigeria may contribute to the low risk of delayed HIV diagnosis observed among students.

We found a progressive decline in the annual total cases of delayed HIV diagnoses over the period examined. Nigeria has no information on national trends in delayed HIV diagnosis in the last decades and studies on trends in the delayed HIV diagnosis in the literature are scarce. The overall percentage of delayed HIV diagnosis observed in this study was consistent with previous studies in Nigeria.^34,35^ Furthermore, studies conducted in other parts of the world, such as Europe and the United States of America, revealed similar trends in delayed HIV diagnosis.^36,37^ Voluntary counselling and testing (VCT) and provider-initiated testing and counselling (PITC) strategies have served as models for HIV testing in Nigeria over the last decade. Several other countries also use these models.^38,39^ Human Immunodeficiency Virus testing is free in Nigeria’s public health facilities,^40^ VCT and PITC (opt-out and opt-in) are available in both public and private clinics and hospitals, as well as via HIV mobile testing.^41^ The improvement in access to HIV testing over the last decade, as well as the testing models adopted in Nigeria, may contribute to the increased rate of testing. This may explain the decline in the annual total cases of delayed HIV diagnosis observed. Despite the easy accessibility of HIV testing, testing often does not occur until years after infection in many cases. While the available HIV testing strategies in Nigeria may contribute to the reduced delayed diagnosis observed over the years of the period we studied, neither of these strategies achieves a high testing rate.^42^

A general belief has been reported in Nigeria that HIV testing and counselling centers are only for HIV positive individuals,^5^ indicating a lack of understanding of the purpose of testing. Many Nigerians fear stigmatization if they are HIV positive.^43^ As a result, patients may refuse HIV testing if offered by physicians. Effective public awareness campaigns may be critical in resolving these misperceptions. Additional support may be required to educate some specific categories of people, such as people at high risk, about the importance of knowing HIV status and early diagnosis. In an effort to address stigmatization, home oral fluid HIV self-testing has been introduced in Nigeria and other African countries, with a reported high acceptability.^44,45^ In a study by Iliyasu et al. 2020, a higher uptake of HIV self-testing was observed compared to other testing strategies among university students in Nigeria.^46^ Self-testing may avoid stigmatization, but positive results need to be linked to care.

The assurance of confidentiality of results may overcome hesitancy to be tested for HIV. Testing for HIV in regular clinics or health facilities with blood draw sent to the laboratory coded without a name may encourage more people to be tested. However, this testing strategy may have a negative effect on referral and linkage to care. All of these testing strategies should be made available not only in primary healthcare centres and other healthcare facilities but also in pharmacies, religious centres, and other community-based organizations. Human immunodeficiency virus testing is an essential component of HIV care. A widespread awareness campaign may play a major role in achieving greater results from the available testing strategies in Nigeria. Promoting HIV testing among all demographic and at-risk groups, including the risk factors observed in this study, such as older ages, retirees, and less-educated populations, will complement these testing strategies. Consequently, effective HIV testing strategies may significantly reduce delayed HIV diagnosis and HIV transmission in Nigeria and other African countries.

Our findings are based on a population tested at a university college hospital in a large city in a single state, and a regional referral center**,** are generalizable to an urban referral population in this location but may not be generalizable to the country as a whole. We examined PLHIV with their first known CD4 count at diagnosis. However, some of the patients may have previous tests without linkage to care. A further limitation is a retrospective design, so risk factors that were not collected could not be analyzed, such as access to testing and perception of the need for testing. The sample size was adequate to detect a statistically significant association between delayed HIV diagnosis and the risk factors.

## Conclusion

Delayed HIV diagnosis is common in the study setting but declining. Delayed diagnoses continue to be a major problem among some demographic groups of the population we studied. While HIV testing has increased in Nigeria over the last decade, the majority of patients are diagnosed at the late stage of infection. Significant expansion of the existing testing strategies, with emphasis on the population at risk is needed in Nigeria to reduce delayed HIV diagnosis. To achieve effective HIV control through care and treatment, a larger portion of PLHIV need to be diagnosed and enrolled in care sooner after they acquire HIV. In addition to more public awareness about the importance of HIV testing, more studies investigating factors responsible for delayed HIV diagnoses should be encouraged in Nigeria. These may lead to a better knowledge of delayed HIV diagnosis and control policy.
